# Mathematical-based microbiome analytics for clinical translation

**DOI:** 10.1016/j.csbj.2021.11.029

**Published:** 2021-11-22

**Authors:** Jayanth Kumar Narayana, Micheál Mac Aogáin, Wilson Wen Bin Goh, Kelin Xia, Krasimira Tsaneva-Atanasova, Sanjay H. Chotirmall

**Affiliations:** a Lee Kong Chian School of Medicine, Nanyang Technological University, Singapore, Singapore; b Biochemical Genetics Laboratory, Department of Biochemistry, St. James’s Hospital, Dublin, Ireland; c Clinical Biochemistry Unit, School of Medicine, Trinity College Dublin, Dublin, Ireland; d School of Biological Sciences, Nanyang Technological University, Singapore, Singapore; e Division of Mathematical Sciences, School of Physical and Mathematical Sciences, Nanyang Technological University, Singapore, Singapore; f Department of Mathematics & Living Systems Institute, College of Engineering, Mathematics and Physical Sciences, University of Exeter, Exeter EX4 4QF, UK; g Department of Respiratory and Critical Care Medicine, Tan Tock Seng Hospital, Singapore

**Keywords:** Microbiome, Integration, Mathematical modelling, Microbial association analysis, Topological data analysis, Machine learning

## Abstract

Traditionally, human microbiology has been strongly built on the laboratory focused culture of microbes isolated from human specimens in patients with acute or chronic infection. These approaches primarily view human disease through the lens of a single species and its relevant clinical setting however such approaches fail to account for the surrounding environment and wide microbial diversity that exists *in vivo.* Given the emergence of next generation sequencing technologies and advancing bioinformatic pipelines, researchers now have unprecedented capabilities to characterise the human microbiome in terms of its taxonomy, function, antibiotic resistance and even bacteriophages. Despite this, an analysis of microbial communities has largely been restricted to ordination, ecological measures, and discriminant taxa analysis. This is predominantly due to a lack of suitable computational tools to facilitate microbiome analytics. In this review, we first evaluate the key concerns related to the inherent structure of microbiome datasets which include its compositionality and batch effects. We describe the available and emerging analytical techniques including integrative analysis, machine learning, microbial association networks, topological data analysis (TDA) and mathematical modelling. We also present how these methods may translate to clinical settings including tools for implementation. Mathematical based analytics for microbiome analysis represents a promising avenue for clinical translation across a range of acute and chronic disease states.

## Graphical abstract

**Figure:**
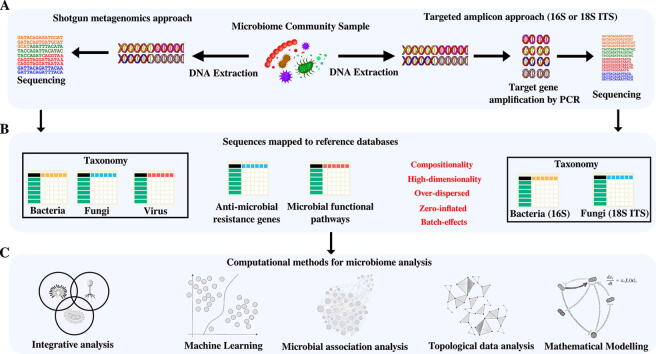

## 1 Introduction

### 1.1 The human microbiome and its role in health and disease

The human microbiome represents a complex and dynamic ecosystem, now established as an important clinical correlate of health and disease [1]. With increasing characterization of the microbiome, our understanding of microbial pathogenesis has progressed exponentially, evolving from focused analysis of individual pathogens to a more holistic analysis incorporating ecological concepts such as diversity, community, and species interaction [2]. The human microbiome is primarily composed of bacteria, viruses and fungi, all of which dynamically interact in a complex manner necessitating multi-dimensional analytic approaches. Such approaches must adapt iteratively as we gain deeper insight into novel aspects of the microbiome’s functionality [3], [4], [5], [6]. The development of analytical pipelines and mathematical models is therefore critical as it permits deeper exploration of the microbiome towards better clinical insight and potential translation [5], [7]. Leveraging upon seminal studies in the gut, human microbiome studies now include multiple anatomic sites and major scientific initiatives such as the human microbiome project have provided a strong base from which the field has evolved [1], [3]. Our existence as ‘holobionts’, composed of human and microbial cells is now clearly established and many physiological processes associate with microbiome composition including digestion, immune regulation and detoxification [1]. Given the increasing appreciation of the centrality of microbes to critical human functions, it is unsurprising that illness and disease is accompanied by significant shifts in microbial composition [8]. Next-generation sequencing (NGS) to derive microbiomes is therefore being increasingly applied across medical disciplines in large observational studies, endophenotyping efforts and clinical trials. Interrogating the generated data requires a careful application of appropriate analytical techniques, and mathematical-based microbiome analytics has emerged as an important means to uncover important signals that may possess potential for clinical translation.

### 1.2 Next-Generation Sequencing (NGS): Targeted amplicon & metagenomics

**Fig. 1:**
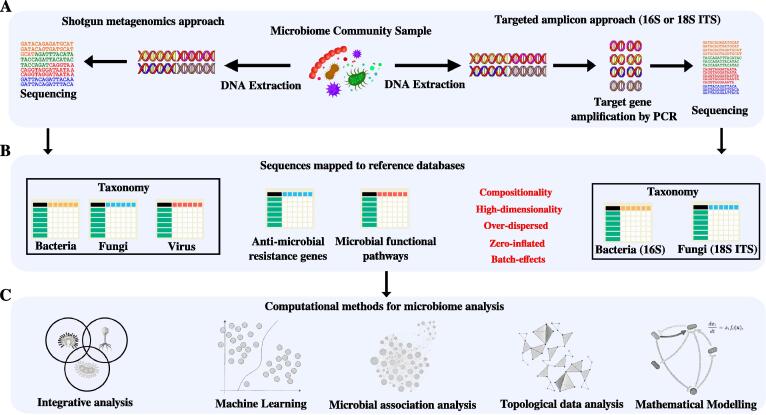
Overview of the analytical approaches to microbiome data. (A) Microbiome community samples can be assessed by (1) whole genome shotgun metagenomics: where the whole DNA content is sequenced or (2) Targeted amplicon sequencing: where a targeted region (i.e. 16S in bacteria or ITS in fungi) is amplified by polymerase chain reaction (PCR) followed by sequencing. (B) The derived sequences are next mapped to reference databases to yield taxonomic, anti-microbial resistance or functional profiles of the microbiome (whole genome shotgun metagenomics) or taxonomic profile (targeted amplicon sequencing). Derived microbiome profiles suffer from compositionality, high-dimensionality, over-dispersion, sparsity, and batch effects. (C) Various computational approaches for microbiome analytics can be leveraged including integrative microbiome analysis, machine learning, microbial association analysis, topological data analysis and mathematical modelling.

Due to the rapid advances in microbial DNA sequencing, our understanding of the human microbiome has rapidly shifted from a microbe-centric, culture-based approach (requiring *a priori* assumptions about the type of sample or disease under investigation) to a less biased pathogen-agnostic NGS approach using a variety of sequencing techniques. Most studies employ targeted amplicon sequencing of the 16S ribosomal RNA gene, one that allows a taxonomic identification of bacteria [9] (Fig. 1A). By corollary, targeting conserved elements of the fungal ribosome, the internally transcribed spacer region (ITS), the fungal microbiome (the mycobiome) can be evaluated, while the analysis of the virome has been challenging and less well defined [10], [11]. The current microbiome literature remains heavily biased toward characterisation of the bacteriome, although there is an increasing awareness of the significance for both fungal and viral kingdoms in determining overall composition and function, including the potential for intra-kingdom interaction necessitating integrated analysis [6], [10], [11], [12]. Whole-genome shotgun (WGS) metagenomics provides a less biased and more holistic alternative to targeted amplicon sequencing and is being increasingly employed in microbiome research (Fig. 1A). While less biased and less susceptible to PCR-associated background contamination, metagenomics is capable of functional profiling although does provide challenges for low biomass samples or samples containing high levels of background human DNA (Fig. 1B). Lower abundance organisms including fungi may be underrepresented or potentially undetected by metagenomics despite serving important biological roles. Here, application of tailored sample preparation methods or blended WGS-target amplicon sequencing approaches may be required to accurately capture true microbial composition. Pacific Biosciences Single Molecule Real Time (SMRT) and Nanopore sequencing protocols further represent ongoing areas of research which promise to bring improvements including full length 16S rRNA gene sequencing and strain-level genomic comparisons but await application in large-scale clinical studies [13], [14], [15]. A further important consideration is the analysis of RNA over DNA (meta-transcriptomics) which better reflects metabolically active microbes and identifies RNA viruses [16], [17]. Notwithstanding such considerations, and the potential pitfalls inherent to the data engineering phase (reviewed in depth elsewhere [9]), the main output of microbiome sequencing workflows remains a set of individual compositional microbiome profiles that serve as a starting point for downstream analysis (Fig. 1B).

### 1.3 The compositional challenge of microbiome data

In 1897, a classic paper by Karl Pearson indicated the dangers of computing correlations between ratios ($\frac{x_{i}}{y}$) with common denominators [18]. This paper implied that correlation analysis of compositions $\left(\frac{x_{1}}{y},\frac{x_{2}}{y},\cdots ,\frac{x_{n}}{y}\right)$, where the ratios ($\frac{x_{i}}{y}$) are subjected to a sum-constraint $\sum_{i}^{n}\frac{x_{i}}{y}=constant$; may lead to spurious correlations when there are actually none. Components of a composition are called its parts, and due to its sum-constraint, these samples exist in a mathematical simplex i.e. a 3-part composition resides in a triangle, 4-part in a tetrahedron and so on for higher-dimensional simplexes (see Section 2.4). For example, the relative abundance of n microbial species in a biological sample resides in a n-dimensional simplex space as opposed to an n-dimensional Euclidean space. Most statistical models assume independence between features which does not hold true for compositional data due to its inherent dependency between features. Conventional multivariate data analysis techniques including the Pearson/Spearman correlations, Euclidean distance and multivariate comparisons were developed for data that reside in a Euclidean space and hence not applicable to compositional data. Historically, confusion surrounds compositional data analysis and improper statistical methods have been applied [19]. To address this problem, the Compositional Data Analysis (CoDA) framework was initiated by Aitchison in the 1980s and was based on the theory of log-ratios [19]. This framework has now further extended to include rigorous statistical approaches to analyse compositional datasets [20].

Microbiome datasets derived from next-generation sequencing (NGS) inherit its technical and analytical limitations. In particular, the issue of “normalization” requires consideration given constraints of sequencing capacity i.e., the total number of read counts in a single NGS run. Further, beyond a certain point, the number of reads (or read depth) obtained is irrelevant as it is derived from a random sample of size-selected DNA fragments bound to a sequencing flow cell in accordance with their relative molarity, thus rendering microbiome datasets compositional. Furthermore, each sample does not usually contain exactly the same number of sequence reads and this is attributed to differences in sequencing platforms, experimental difficulties in loading the exact molar amounts of the sequencing libraries and random variation. Hence, microbiome datasets only contain information on their underlying proportions and are often represented as relative abundance, normalized read counts or rarified prior to analysis. Rarefaction involves subsampling of the obtained read counts to a common read depth, however its use is questioned as it leads to loss of potentially useful information [21]. Apart from the statistical complications of microbiome datasets due to their compositionality, the problem is further compounded by high dimensionality and sparsity, as the microbiome comprises several types of microbes and large zero values (Fig. 1B. Hence, studies that use traditional methods to normalize microbiome data, rather than CoDA based methods, may miss important clinical insight due to suboptimal data normalization protocol. For instance, the centered log ratio (CLR) transformation is often used in CoDA based analysis for microbiomes. Given the count vector of ‘D’ taxa’s in a sample $x=\left[x_{1},x_{2},\cdots ,x_{D}\right]$, the CLR transformation of the sample is defined as, $x_{\mathrm{clr}}=\left[log\left(\frac{x_{1}}{G\left(x\right)}\right),log\left(\frac{x_{2}}{G\left(x\right)}\right),\cdots ,log\left(\frac{x_{D}}{G\left(x\right)}\right)\right]$, where $G\left(x\right)$ is the geometric mean of x. The clr- transformed values are scale-invariant, i.e. the same ratio is expected to be obtained in a sample with few read counts or with many read counts, only the precision of the estimate is affected [20].

Compositional methods themselves however also suffer from loss-of-scale issues, and therefore efforts toward absolute quantification such as ‘spike in’ approaches have been employed. This involves the addition of exogenous pre-determined microbial material, that act as an internal control, to derive the absolute abundance of a microbe by ‘back-normalization’ which mitigates the compositionality of microbiome datasets. These methods however remain under-utilized and largely dependent on the exogeneous additive material. Interestingly, recent work describes the use of a cell-based multi-kingdom spike-in method (MK-SpikeSeq) to derive absolute abundance, and applies conventional mathematical modelling techniques (see Section 2.5) to derive precise community dynamics of the microbial ecosystem, otherwise not possible using compositional datasets [22].

### 1.4 Batch effects and the microbiome

Batch effects represent unwanted variation in data caused by factors unrelated to the one of interest, for instance variable experiment times, handlers and reagent lots [23]. Such unwanted effects are endemic in high-throughput methods such as NGS that remain limited by factors such as sequencer capacity, multiple handlers, sample collection, storage or bioinformatic pipelines. Correcting for such effects is imperative, as they otherwise obscure true biological phenomena, reduce statistical power, reproducibility, generalizability or even potentially create artefactual effects [23]. Several Batch Effect Correction Algorithms (BECA) exist but their effectiveness is poorly understood and if inappropriately implemented may lead to loss of biological variation and inflate false positive and false negative rates [24].

Currently, BECAs for microbiome datasets are under-developed and largely derived from gene-expression analysis [25], [26]. Batch effects in microbiome data are usually managed by first transforming the dataset using log transformation approaches such as the Centered Log Ratio (CLR) to account for compositionality and sparsity, followed by standard batch correction methods such as ComBat, Batch Mean Centering (BMC) and Surrogate Variable Analysis (SVA), if their assumptions are satisfied [25]. Specifically, for case-control microbiome studies, model-free percentile normalization methods may be implemented for batch effect correction [26]. Limitations to the currently available microbiome batch correction strategies include the often erroneous assumption that a data transformation alone will satisfy the strong assumptions of batch correction methodologies [25]. Additionally, batch-correcting datasets with different microbial community proportions and other imbalances may result in a misestimation of batch-associated variances [27]. To avoid errors due to misassumptions of data normality, non-parametric or distribution-free methods with the ability to accommodate microbiome data characteristics are required. Ideally, such methods must be both effective and precise in targeting batch effects while preserving biological variation [28]. Furthermore, they should be able to cope with batch effects across microbiome studies without being restricted to one type of experimental design (e.g., case–control studies for percentile normalization). Importantly, batch effect-resistant methods such as Similarity Network Fusion (SNF), which mitigate batch effects between -omic datasets (including microbiomes) by creating -omic specific similarity networks prior to merging (see Section 2.1.1) will likely become increasingly relevant in future work and should be considered when developing new analytical methodologies for microbiome analysis.

## 2 Emerging computational methods for microbiome analytics

### 2.1 Integrative analysis

Owing to the rapid progress in NGS, we are now able to identify a pool of microbes from human specimens and characterise their taxonomical, functional and resistome profiles [29] (Fig. 1B. Beyond bacteria, fungi, viruses, and their corresponding bacteriophages all represent important components of the human microbiome however most work to date has largely focused on bacteriomes [29]. The key reasons for such bias include a lack of strong reference databases for the viral and fungal kingdoms but also a lack of integrative strategies and computational pipelines to holistically assess intra-kingdom microbiomes. In addition, microbes rarely exist in isolation and usually form complex, interactive, interkingdom communities that encompass the human holobiont [8]. Consequently, a holistic integrative ‘multi-biome’ approach is most appropriate to accurately represent the true physiological *in vivo* state and gain a greater understanding of any underlying disease pathology.

The development of integrative microbiome analytics has been slow and its progress largely dependent on established integrative -omics methodologies as applied to genomic, transcriptomic, proteomic, epigenomic and metabolomic data [6]. Although microbiome datasets are comparable to other omics, the integration of multi-biome datasets using appropriate analytical methods must be carefully considered before implementation, as such analyses may be influenced by artefacts of the ‘omics’ technologies itself. The following sections discuss multi-omics integration methodologies that may be applied to microbiome data.

#### 2.1.1 Similarity network Fusion (SNF)

Similarity Network Fusion (SNF) is a network-based multi-omic data integration method that has been successfully applied to microbiomes [12]. For each respective omic dataset, SNF first creates a similarity network using an appropriate measure of similarity. This is next followed by normalization of cross-network similarity scores for individual datasets before merging to create an integrated network that can be applied clinically. Integrated ‘patient networks’ can then be assessed to classify or identify clinically relevant subgroups based holistically on integrated ‘multi-omic’ data [6]. SNF provides increased cluster robustness and accuracy, down-weights 'noise' and increases statistical power to detect rarer subgroups from relatively small cohorts. It handles heterogeneous and missing data well however has limitations including its assumption of equal weights (to each integrated dataset) and use of a single similarity metric to capture what is likely complex biological phenomena. Several more recent SNF based methods have been developed to address the various limitations of traditional SNF [6]. Recent work from our group has developed and applied weighted Similarity Network Fusion (wSNF), a method that allows ‘weightage’ of each individual dataset used in the integration process. We applied wSNF to integrate bacterial, viral and fungal microbiomes in bronchiectasis which resulted in the identification of clinically relevant ‘high-risk’ patient groups with increased precision as compared to use of single kingdom microbiome datasets [12]. This work serves to underscore the advantage of microbiome data integration in deriving clinically meaningful insights as opposed to single kingdom views of the microbiome.

#### 2.1.2 Data integration analysis for Biomarker discovery using latent cOmponents (DIABLO)

DIABLO is a supervised multi-omics integration strategy developed as part of the mixOmics framework [30]**.** This supervised integrative approach is based on the Generalized Canonical Correlation Analysis that aims to maximize correlations between low-dimensional projections of the input multi-omics datasets. DIABLO improves this by accounting for sparsity of the omics’ dataset, a feature particularly relevant to microbiome data and additionally supervises these low-dimensional projections to explain categorical outcomes of interest. Specifically, DIABLO employs discriminant analysis to identify co-expressed or co-related omics features across datasets that may explain the outcome of interest. Importantly, DIABLO assumes linearity, provides no information on causality, and captures only linear relationships which may not hold true in the context of microbiomes. DIABLO also cannot account directly for batch effects that arise across included datasets due to inherent differences in the experimental platforms and analytical pipelines used for each respective dataset [31]. DIABLO has been successfully applied to integrate gut microbiomes with metabolomics, clinical data and microbial function, and this increases classification accuracy compared to singular data analysis, serving to further highlight the analytical gains derived from data integration approaches [32].

#### 2.1.3 Multi-Omics Factor analysis (MOFA)

Multi-Omics Factor Analysis (MOFA) represents an un-supervised statistical framework for multi-omics data integration including microbiomes [33]. MOFA can be considered a generalization of Principal Component Analysis (PCA) that assesses multiple omics datasets as the primary input, with the aim to identify common latent low-dimensional representation of the data. MOFA captures common variation across the various datasets and highlights contributions through feature weights. Important limitations of MOFA include the inability to appropriately capture non-linear relationships and the assumption of independence between features, which in particular may not hold true for microbiomes as microbes exists in communities [34]. MOFA has been employed to integrate bacterial, viral, and fungal components of the intestinal microbiome in critically ill patients with and without sepsis, prior to and following antibiotic exposure. These analyses reveal a modulation of gut microbiomes that involve interkingdom interactions, where overgrowth of potentially invasive viral and fungal organisms is driven by changes to the bacteriome [33].

### 2.2 Machine learning and microbiomes

Machine Learning (ML) is a class of algorithms that mimic human learning by detecting patterns in data. ML algorithms predict and make decisions without being explicitly programmed but instead use the patterns learnt from the “training data”. The use and development of ML algorithms is rapidly accelerating with emerging applications across multiple domains including the practice of medicine. ML methodologies can be categorized into two schemes: (1) supervised and (2) unsupervised. Supervised algorithms learn patterns that map the presented input data to the desired output, given by ‘labelled data’, using *a priori* identified features for classification and/or regression. In contrast, unsupervised algorithms are only presented with input data and no labelled data, leaving it to find patterns or discover groups in the input dataset which includes clustering. Therefore, a general ML workflow involves (1) data preparation, (2) feature selection, (3) choosing a ML model, (4) training the model, (5) model evaluation, (6) parameter tuning and (7) model testing. As applied to microbiome datasets, ML techniques can have far-reaching benefits for host-trait or disease prediction including risk stratification [35]. The use of ML methods for microbiome-based prediction and classification has been investigated in several studies, with some developing their own framework including DeepMicro, MetaML, Phy-PMFRI, mAML and PopPhy-CNN [36], [37], [38], [39]. Machine learning based models have been extensively applied to large-scale microbiome studies with varied applications including the prediction of habitual diet, disease sub-phenotyping and in the identification of ‘enterotypes’ through clustering and bio-marker identification, for instance linking visceral fat to gut microbial composition [40], [41], [42].

#### 2.2.1 DeepMicro

DeepMicro represents a ML framework for microbiome-based prediction. This framework first represents high-dimensional microbiome datasets into low-dimensional latent space followed by implementation of ML models. DeepMicro provides algorithms for dimension reduction such as principal component analysis (PCA), random projection and ‘Auto Encoders’: a class of un-supervised deep learning algorithms capable of learning representation in terms of latent features and with the potential to capture non-linearities [36]. Following this, DeepMicro provides the user options to implement ML algorithms such Random Forest, Support Vector Machine and Multi-Layer Perceptron algorithms for predictions based on the learned latent representation of the dataset. This framework also employs a thorough k-fold cross-validation scheme for hyper-parameter optimization. It has been established (using five different diseased microbiome datasets) that DeepMicro outperforms other ML frameworks for disease prediction, while significantly reducing dimensionality and training time by 8- to 30-fold suggesting value for microbiome analytics [36].

Critically, however, there are at present only few ML-based microbiome studies that have produced direct clinical translation [35], [43]. There are several reasons to explain this: a lack of quality data or sufficient sample size, inadvertent fitting of confounders, lack of generalizable models (i.e. the model does not work beyond the training data) or explainability (i.e. the model cannot be mined for clinical insight), an under-representation of healthy individuals (or non-representativeness in the training set) and heterogeneity of disease phenotypes (hidden sub-labels are present and may confound proper learning), all of which are further complicated by the compositionality and high dimensionality of the underlying datasets. Such limitations may be partially addressed by implementing appropriate data preparatory steps to account for compositionality, batch effects and confounders [44], [45]. Further, intelligent feature engineering by combining other techniques such as microbial association networks, mathematical modelling or topological data analysis all hold promise in providing better resolution and robustness for these ML models. Importantly, there remains an unmet need for explainable ML models that support researchers understanding of the underlying biological phenomenon rather than the current provision of only accurately predicting disease classes [46]. Novel and emerging methods including alignment-free (i.e. taxonomy free) approaches that can be leveraged from metagenomic datasets are being increasingly explored and will likely be a focus of future ML applications in the translational space avoiding the bias of current taxonomy-driven approaches [47], [48].

### 2.3 Microbial association analysis

Most biological conditions cannot be attributed to an individual organism but rather a specific microbial signature, consisting of multiple microbes that illustrate the complex underlying ecology that includes microbial interactions [12], [22], [33]. Network science is therefore recognized as an important technique, for the analysis of complex systems such as that of human microbiomes. Many fundamental discoveries and applications utilize network theory at their core. This includes the Google search algorithm, the discovery of emergent phase transition in material science and the invention of reference models for the internet [49]. In the context of human microbiomes, network science is used to construct microbial association networks [50]. Increasingly, a significant number of methods are being developed for improved microbial network analysis, where microbial clades are represented as nodes and edges (between them) determine associations [51]. A summary of several emerging methods for microbial network inference are detailed below.

#### 2.3.1 Co-occurrence network analysis including renormalization and bootstrap (CoNet)

CoNet is an ensemble-based network inference algorithm producing a weighted undirected graph developed specifically for microbiome datasets [52]. The algorithm uses multiple similarity measures such spearman, kendall and pearson correlations, Kullback–Leibler divergence and bray-curtis similarity to identify consensus microbial similarity networks. Importantly, spurious correlations due to compositionality is accounted for by bootstrapping and renormalization approaches (ReBoot) [53]. The algorithm is available as a Cytoscape plugin and therefore lends itself to implementation for individuals familiar with the Cytoscape suite of functions [52]. CoNet has successfully identified interactions between microbial species in interstitial fibrosis, and is also among pathobionts associated with cancer cachexia [54], [55]. Implementation of CoNet with general boosted linear models offers directionality to these microbial associations hinting at causal direction, however, does not establish accurate causality and/or causative mechanisms [53].

#### 2.3.2 Sparse inverse covariance estimation for ecological association inference (SPIEC-EASI)

Spiec-Easi leverages the concept of conditional independence to identify underlying undirected microbial networks by assuming sparsity and estimating the invertible covariance matrix. It employs data transformations developed for compositional data to account for compositionality. Graph creation can be performed by two methods: sparse neighbourhood (which creates a graph node-by-node) and inverse covariance selection (that creates the entire graph) [56]. Finally, stability-based model selection is conducted to infer optimal sparsity of the derived microbial network [56]. Spiec-Easi has been successfully applied to identify cross-kingdom interactions between bacteria and fungi, and to identify key species and assess the topological properties of microbial association networks in antibiotic treated mice [57], [58].

#### 2.3.3 Microbial dynamic systems inference engine (MDSINE)

MDSINE is a suite of algorithms using microbiome time series data to infer dynamic system models, which in turn, extrapolate the directed weighted microbial association networks and associated perturbation effect networks [59]. The algorithm takes in two inputs: a temporal microbial abundance and temporal microbial biomass represented for instance by universal 16S rRNA quantitative PCR. This technique accounts for compositionality by estimating non-compositional microbial growth concentrations and their temporal changes using the input datasets. Derived data is then used to infer the parameters of the dynamic system, such as generalized Lotka Volterra models (section 2.5.1). These parameters are then used to infer microbial association networks [59]. MDSINE, in murine *Clostridium difficile* infection models, has been used to determine causal interactions, microbial dynamics, predict stable subcommunities, and to identify microbes most crucial to the integrity of the community under perturbation [59].

Identifying causal relationships between different taxa, including those between the microbiome and other -omics, such as metabolites, remains crucial for understanding the biological mechanisms underlying host-microbe interactions. Clinical translation of microbiome analysis therefore requires the biological mechanisms of interaction between host and microbe to be well understood, essential to make reliable predictions medically and to explore appropriate intervention strategies that have a microbial focus. Experimental methods are therefore most optimal, effective and accurate for establishing such causal relationships [51]. Nevertheless, network inference methodology that generates directed networks using longitudinal datasets such as MDSINE, LSA (Local Similarity Analysis) and TIME (Temporal Insights into Microbial Ecology) can reliably infer causal relationships between taxa [51]. Furthermore, mathematical modelling techniques (Section 2.5) such as genome-scale metabolic modelling along with flux balance analysis may reveal causal interactions between microbes and their hosts [60], [61]. These techniques coupled to multi-omics may be used to examine the dynamics of the modelled system in addition to inferring relationships between taxa and their omic-features [62], [63], [64]. Advanced analytical methods such as these may then be used to shortlist for instance key relationships for subsequent experimental validation.

Important limitations of such network analysis however include dependence of microbial association networks on sampling resolution, limitations of sequence read analysis and the inability to distinguish live, dead, or dormant cells, although the latter may be accounted for by use of RNA sequencing techniques [50]. Concerns with the interpretability of the output networks remain an important challenge for those using such analysis [50].

### 2.4 Topological data analysis (TDA) models for microbiomes

Assessment of key microbial taxa is performed by assessing their interaction with other microbes within a community. Microbial association analysis results in microbial networks of interacting taxa (or nodes). Studying and quantifying these structures and associated patterns of the network using graph measurements remains important to identify key components (taxa) of the microbial network. Graph measurements such as degree, stress centrality and betweenness centrality have all been used and shown to have potential clinical translatability and significance [12], [65]. In view of the potential clinical utility observed from studying network structures, the field of computational topology, concerned with the study of shape and connectivity, holds significant potential to advance current microbiome analytics.

Topological data analysis (TDA), a widely-used dimensionality reduction and featurization approach is used to study the “shape of data” using models and methods from computational, combinatorial and algebraic topology [66]. Since high dimensional data cannot be directly visualized, TDA may be leveraged to infer topological aspects. TDA methodologies are therefore based on (1) data representation with topological models, including simplicial complexes and hypergraphs and (2) data characterization with topological invariants, including Betti number and Euler characteristics among others. A key feature of TDA is to generalize graphs and networks to simplicial complexes. Physically, edges in networks (or graphs) characterize pair-wise interactions, while complexes, key components of a simplicial complex, characterize higher-dimensional interactions, such as many-body interactions (Fig. 1C). Given the success of network theory in its application to medicine and biology, simplicial complexes and their measures represent a promising avenue for translational research and clinical application. Another key concept in TDA analysis remains topological-invariant, which is a topological measurement that is invariant under continuous deformation. Topological invariant measures represent the most intrinsic and fundamental properties of structures. Among all topological invariants, the most commonly used is the Betti number, which ranks homology groups. Geometrically, dimensionally different Betti numbers represent different homology generators, including their connected components such as loops, circles and holes [66]. Persistent homology is therefore a key model in TDA, providing a bridge between geometry and topology, and in study of the “birth” and “death” of homology generators during a filtration process. Mathematically, the persistence of homology generators encode the geometric information of structures. TDA may derive new insights and deepen our current understanding of microbiome data although the only TDA algorithm applied thus far to microbiome research is Mapper, an approach that aims to uncover and visualize the topological properties of microbiomes [67], [68], [69], [70].

Mapper is a computational method for extracting simple descriptions of high dimensional datasets in the form of simplicial complexes. Mapper transforms high-dimensional microbiome profiles into simplicial complexes that reflects geometric aspects i.e., microbiome variation across samples. Network nodes represent a set of samples with similar microbiome profiles and links describe the intersection of samples between two or more nodes. Mapper accomplishes this in three distinct steps: (1) dimension reduction, (2) covering, and (3) clustering. Dimension reduction is performed by projecting high-dimensional data points (representing microbiome profiles) to a low-dimensional space using user-defined functions called ‘filter’ functions. Filter functions can also be referred to as ‘lenses’ because the selection of such functions potentially reveal different aspects of the dataset. For example, Shannon diversity and Berger-Parker dominance indices act as ‘filter’ functions that represent the sample based on its taxonomic diversity and taxonomic dominance as views, which are not necessarily the same. Upon reducing dimension, Mapper divides this low-dimensional space into several covers of equal size overlapping with one another. Covers in this space capture local neighbourhoods of data, and the overlap connects these neighbourhoods to capture global structure. Finally, Mapper implements clustering on the pull-back of each cover, to group samples with similar microbiome profiles. This step is critical to retain the original distance information of the high-dimensional microbiome profile as information concerning original distances between samples may be potentially lost after dimension reduction. For example, two samples that are far apart in high-dimensional space (i.e. dissimilar in microbiome profile) might be projected as close neighbours in the low-dimensional space (e.g., due to similar diversity values). The simplicial complex is then generated such that each node represents a cluster, and a link is then drawn between nodes if they share common samples within their clusters.

The adoption of TDA based techniques (such as Mapper) to microbiome datasets has now led to frameworks such as tmap which recently illustrates superiority in detecting non-linearities in data, for instance in enterotype analysis, driver species identification, and microbiome-wide association analysis in conjunction host metadata [70]. Mapper has been successfully used to uncover state transitions in human gut microbiomes and asthma endotypes based on microbiome profiles [67], [68], [69]. Mapper importantly outperforms PCA and PCoA in distinguishing patient characteristics based on microbiome profiles [71].

### 2.5 Mathematical modelling of the microbiome

Most microbial association analyses can identify significant dependency between microbes within the microbiome however such dependencies cannot predict ‘causality’ and a system’s future behaviour. Despite this, the identification of such dependency may be used to build dynamic mathematical models of the microbial community that may be used to assess causality, i.e. the effects of its different components on one another and make predictions about its behaviour. In addition, mathematical models are constructed based on the understanding and/or assumptions of the system’s mechanisms, hence, are invaluable in studying a system’s behaviour to changes in its parameters and/or validating assumed mechanisms through experimentation. The power of explainability; inherent to mathematical models can also be used as a complementary strategy along with data analysis, that aims to extract information from data for clinical application. However, thus far, few studies have attempted to apply such modelling techniques to microbial communities [62]. Within a mathematical model, the modelling units themselves represent the most basic interacting entities of the overall system; for instance, taxa (or microbial species), individual cells, functional guilds, or the overall community. The choice of unit dictates model resolution, the subsequent simulated dynamics and defines the potential frameworks that may be employed for analysis. These include the following frameworks: (1) supra-organismal; (2) population-based; (3) heterogenous and/or (4) integrative [62].

#### 2.5.1 Lotka Volterra models

The commonest modelling approach employed in microbiome research remains population-based models such as the generalized Lotka Volterra Models (gLV). These methods study population dynamics of the modelling units and assumes homogeneity of internal states across the individual microbes within each population. Such pairwise models describe potential relationships between ‘n’ species (or taxa) using ordinary differential equations to track their population growth dynamics i.e.:

$$\frac{ds_{i}}{\mathrm{dt}}=s_{i}\left(\mu_{i}+\sum_{j=1}^{n}a_{\mathrm{ij}}x_{j}\right),j=1,\cdots ,n$$

where $s_{i}$ represents species (or taxon) abundance, ‘$\mu_{i}$’ its growth rate and ‘$a_{\mathrm{ij}}$’ the interaction strength between species (or taxa) *‘i’* and *‘j’*. Although this model is widely used and relatively easy to implement, it (1) requires an absolute quantification of abundance to account for compositionality, (2) assumes the additive influence of fitness from pairwise interactions, and (3) does not provide any understanding of chemical intermediates [72]. Alternative modelling techniques of finer model resolution may partially address some of these limitations such as mechanistic modelling, that offers insight into chemical intermediaries between microbes, stochiometric modelling which offers mechanistic insight and compositional Lotka-Volterra models, that allows modelling on compositional data [62], [73]. In addition, thermodynamic models, evolutionary game theory models and integrative modelling strategies may all be potentially useful in clinical microbiomics as they each integrate various other modelling strategies of different resolutions thereby potentially overcoming the inherent weaknesses when only a single approach is used [62].

Generalized Lotka Volterra models have been used to predict gut microbial dynamics in preterm infants accounting for environmental perturbations and revealing microbial blooms [22]. Jones et al. applied gLV models in antibiotic-induced *C. difficile* infection to evaluate microbial dynamics and proposes mathematical model-motivated experiments [74]. Mathematical modelling techniques including community metabolic modelling have been leveraged to derive metabolic networks in the gut and when applied to type 2 diabetes reveals unique network structures that render a significant metabolic influence *in vivo* [75]. To successfully apply mathematical modelling to microbiome analytics, the appropriate choice of model to use is key. Selection should be driven by the intended objective and follow *Occam’s razor* principle i.e. to use the simplest credible model given the biological question under investigation. Such models are characterised by fewest parameters thereby reducing the complexity of the model structure and hence the probability of over-fitting. Available tools such as Akiake Information Criterion (AIC) and Bayesian Information Criterion (BIC) can also systematically and quantitatively compare between models. The mathematical modelling of microbiomes is likely to be an area of significant development for potential clinical translation over the coming years as it allows an understanding of microbial associations under specific conditions, predicts dynamics under fresh conditions and models the outcomes when microbial communities are controlled to perform clinically advantageous functions.

## 3 Summary and outlook

In this review, various emerging mathematical approaches to microbiome analytics have been outlined while emphasizing their potential application in clinical translation (Table 1). Each approach has its strengths however general challenges inherent to microbiome data need to be acknowledged and addressed including compositionality and batch effects. While community ecological techniques including ordination (Principal Coordinate Analysis), diversity, dominance analysis and discriminant taxa analysis such as Indicator species analysis, Similarity Percentages (SIMPER) and Linear discriminant analysis Effect Size (LEfSe) are all commonly employed in microbiome research, a clear need for more advanced models and analytical techniques is now required to match the complexity and rapid development of available NGS approaches. Additionally, current microbiome studies are largely observational and descriptive in nature and there is a clear need for rigorous mathematical and analytical approaches to attain a more precise understanding of the role of microbiomes in human disease. Realizing the underlying biological mechanisms of microbial dynamics, dysbiosis and its interaction with the host is critical for clinical translatability. Considering this, novel yet rigorous mathematical approaches will be necessary to meet the demands to better understand, shortlist ‘causal relationships’ and subsequently engineer microbial communities to impact clinical medicine. It is likeliest that a combination of the presented techniques, applied in an appropriate setting holds significant potential for clinical translation, however, such analytical approaches must be validated in rigorous experimental models to provide confidence in practical applications that may include the engineering of individual species to manipulate a community or as microbial biomarkers in specific disease-states.

**Table 1:** Table summarizing open-source tools and software available for the methods described in this manuscript.

Method	Tools/Softwares available
Compositional Data Analysis	‘compositions’ – a R package [76] ‘propr’ – a R package [77] ‘CoDa Pack’ – a multiplatform standalone software [78]
Similarity Network Fusion (SNF)	‘SNFtool’ – a R package [79] ‘Integrative Microbiomics’ – a webtool for integration of microbiomes [80]
Data Integration Analysis for Biomarker discovery using Latent cOmponents (DIABLO)	Part of ‘mixOmics’ – a R package [81]
Multi-Omics Factor Analysis (MOFA)	‘MOFA2’ – a R package [82]
DeepMicro	‘DeepMicro’ – a python package [83]
Co-occurrence network analysis including renormalization and bootstrap (CoNet)	‘CoNet’ – a cytoscape app [84]
Sparse inverse covariance estimation for ecological association inference (SPIEC-EASI)	‘SpeicEasi’ – a R package [85]
Microbial dynamic systems inference engine (MDSINE)	‘mdsine’ – available as standalone and MATLAB library [86]
Mapper	‘Kepler Mapper’ – a python package [87]

## CRediT authorship contribution statement

**Jayanth Kumar Narayana:** Conceptualization, Methodology, Visualization, Writing – original draft, Writing – review & editing. **Micheál Mac Aogáin:** Conceptualization, Methodology, Writing – review & editing. **Wilson Wen Bin Goh:** Conceptualization, Methodology, Writing – review & editing. **Kelin Xia:** Writing – review & editing. **Krasimira Tsaneva-Atanasova:** Conceptualization, Methodology, Supervision, Visualization, Writing – original draft, Writing – review & editing. **Sanjay H. Chotirmall:** Conceptualization, Methodology, Supervision, Visualization, Writing – original draft, Writing – review & editing.

## Declaration of Competing Interest

The authors declare that they have no known competing financial interests or personal relationships that could have appeared to influence the work reported in this paper.
